# A data-driven approach to stock inhaler programming implementation in Chicago Public Schools

**DOI:** 10.1186/s12982-026-02207-3

**Published:** 2026-06-20

**Authors:** Mariya Patwa, Jennifer Cushman, Rachel Lane, Paige Hardy, Smriti Dhakal, Sean Zolfo, Jeremiah Simon, Jeannette White, TaShunda Green, Molly A. Martin, Andrea A. Pappalardo

**Affiliations:** 1 University of Illinois at Chicago, 840 S. Wood Street, MC 719, Chicago, IL 60612 USA; 2 Chicago Public Schools, Chicago, IL USA

**Keywords:** Asthma, Child health, School health, Health disparities

## Abstract

Asthma rates are high in Chicago, but rescue inhaler access is more limited among Black and low-income populations, which impacts school attendance and academic achievement. Stock inhalers are undesignated asthma inhalers for respiratory distress at school, which may alleviate these needs. We assessed the pre-intervention asthma environment of pilot, ramp-up, and non-pilot Chicago Public Schools to retroactively ensure an equitable scale-up of a stock inhaler intervention across a large urban district. We also examined support plans for asthma by school and student characteristics. We used chi-squared tests, Kruskal Wallis tests, and generalized estimating equation models to analyze the association of asthma status with school and student characteristics within district-run schools. 517 district-run schools and over 266,000 students were included. High schools had a higher median asthma prevalence (6.39%) than elementary schools (4.49%). Students who identify as Non-Hispanic Black had higher odds of asthma than Hispanic students (OR:1.20[1.11, 1.30], *p* < 0.001), but these students subsequently had lower odds of a 504 plan (OR:0.7[0.6, 0.7], *p* < 0.001) and higher odds of an IEP (OR: 1.2[1.1, 1.3], *p* < 0.001) compared to Hispanic students with asthma. Low-income (OR:1.53[1.45, 1.57], *p* < 0.001) and unhoused students (OR:1.43[1.30, 1.57], *p* < 0.001) had higher odds of asthma than those without these characteristics. While the scale-up itself was equitable, the analysis revealed gaps in asthma prevalence and support plans in Chicago Public Schools. Data-driven approaches like stock inhalers in schools are one solution to reducing disparities in access to asthma documentation and medication.

## Introduction

Asthma remains one of the most common chronic conditions among children in the United States, affecting over 4.6 million children [1–3]. They can experience life-threatening exacerbations, steroid-induced side effects, psychosocial disruption, diminished quality of life, and permanent airway changes [4–9]. Uncontrolled asthma increases absenteeism, worsens achievement test performance, increases risk of less favorable student evaluations by teachers, interferes with daily functioning, and imposes significant financial burden [10–12].

Pediatric asthma is a significant public health concern in Chicago where 16% of families have at least one child with asthma [13]. Non-Hispanic Black and/or low-income children experience disproportionate rates of emergency department (ED) visits and hospitalizations, driven by factors such as under-diagnosis, environmental triggers, and limited access to preventive and rescue medications [14, 15]. These disparities have been recognized in Chicago Public Schools (CPS), but recent data is limited, especially in the context of recent asthma school-based interventions [16–21].

Access to rescue inhalers during school hours is a critical yet often unmet need. While the US recommends that children store a personal inhaler at school and/or self-carry a personal inhaler with them at all times, schools must address emergency situations for children in respiratory distress who are undiagnosed, forget their inhalers, or run out of medication [20, 22, 23]. A practical solution is to keep a supply of stock inhalers and disposable spacers available in schools for use by any student experiencing respiratory distress [24]. While not mandatory or available on the national level, stock inhaler programs increase access to rescue inhalers, enable students to safely return to class, and may decrease the need for 911 calls to schools for asthma, improving both student health and attendance [25].

Illinois Public Act 100-0726 was passed in 2018, which authorized the use of “stock” or undesignated asthma medication for use in emergencies in schools [26]. Implementation was initially slow, with only four reports of undesignated asthma medication administration during the 2018–2019 school year (SY) [27–29]. The RESCUE-IL program secured $2.4 million in funding to support statewide stock inhaler programming to Illinois public schools [30]. As of March 2024, RESCUE-IL provided medication, equipment, training, reporting and educational support to over 3,100 schools, representing approximately 80% of Illinois public schools.

Implementing a large stock inhaler program in CPS, presented unique challenges given the district’s size and diversity. To prepare, the research team collaborated with CPS from 2016 through 2021 to identify key needs and feasibility factors. A limited pilot with four schools began in September 2023, followed by a ramp-up to 306 schools by the end of 2024. However, the initial pilot sites were selected based on available asthma-related 504 plans (which provide accommodations to ensure equal access to education without specialized instruction) involving asthma alone [31]. At this time, we did not have comprehensive district-level data to inform equitable implementation. Understanding the pre-implementation asthma environment is therefore essential to evaluate whether resources were distributed equitably and to guide ongoing dissemination.

The purpose of this manuscript is to present the pre-implementation CPS asthma environment data to assess the equity of the CPS stock inhaler program implementation and subsequent dissemination. Specifically, we sought to (1) characterize the asthma environment in relation to pilot, ramp-up, and non-pilot district schools, and (2) examine access to asthma-related accommodations across school and sociodemographic factors. We hypothesized that disparities in the asthma environment exist in pilot versus ramp-up schools and that district-wide disparities remain present across CPS schools.

## Methods

### Study design

This is a retrospective secondary data analysis of student and school-level demographics from the 2021–2022 and 2022–2023 school years (SY). This study was approved by our Institutional Review Board and the CPS Research Review Board prior to data collection.

### Chicago Public Schools (CPS)

CPS is a large public school district in the US, serving over 300,000 students across more than 600 schools. CPS reflects a student population where 46% of students identify as Hispanic and 36% as Black [32]. Given its size and demographics, CPS serves as a benchmark for comparison with major urban school districts.

### Inclusion/exclusion criteria

In the pre-implementation phase (2022–2023), pilot schools were selected based on three criteria: (1) asthma prevalence, indicated by the number of asthma 504 plans per CPS policy (not by number of Asthma Action Plans (AAPs) or indicated diagnosis of asthma on state-based yearly physical forms), (2) nurse coverage available five days a week, and (3) school principal willingness to participate [31]. Schools during the subsequent ramp-up were chosen alphabetically.

### Pilot and Ramp-up Schools (Fig. 1)

**Fig. 1:**
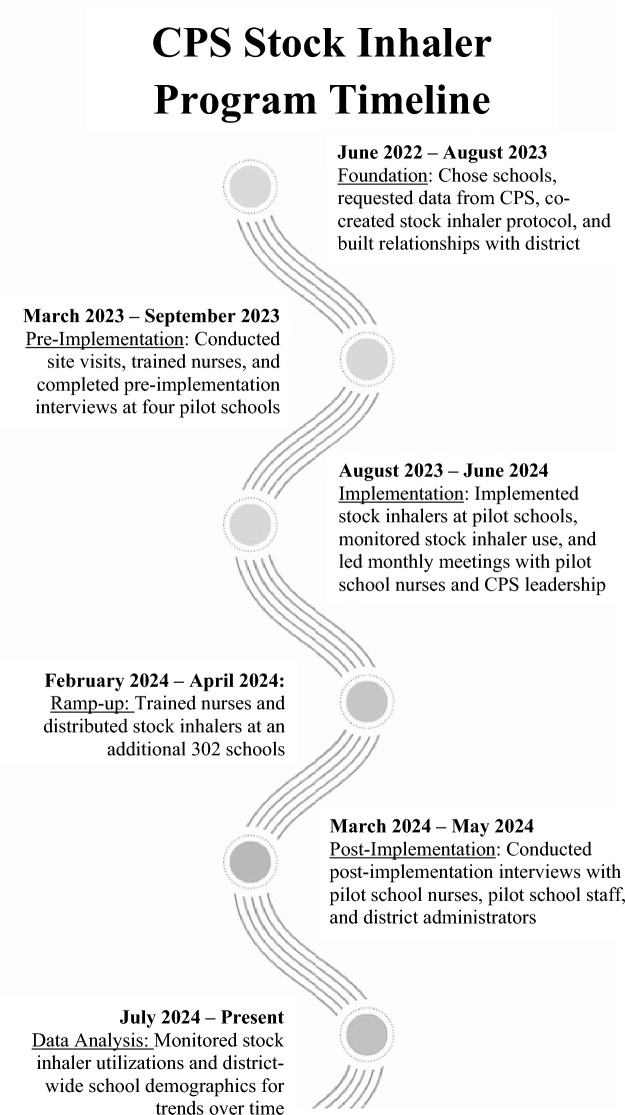
CPS Stock Inhaler Program Timeline. This figure outlines the timeline for the implementation of the CPS Stock Inhaler Program, which started in June 2022 with foundation building, then progressed through the pre-implementation, implementation, ramp up, interview, and data analysis phases

In February 2024, the program expanded to 100 schools. Then in April 2024, school participation expanded to 306 schools (Phase 1). By December 2024, all 622 schools were included (Phase 2). However, due to different leadership and reporting structure of CPS, only 517 district-run schools for which data was available will be presented in this manuscript.

### Study variables

This study relies on student data obtained from the school district data team obtained by a standing data use agreement with our institution and delineated by a specific scope of work for this project. Data included student counts, aggregated by school and sociodemographic, health, and educational characteristics. The terms “school characteristics” and “school-level variables” interchangeably refer to the variables of region, grade level, school size, and attendance rate. School size and attendance rate categories were determined by approximate tertiles. The terms “Student Characteristics” and “student-level variables”, interchangeably refer to gender, race and ethnicity (reported as a composite variable by CPS), asthma diagnosis, food allergy (FA) and other allergy, Individualized Education Program (IEP, which is a more intensive accommodation plan that modifies the school curriculum) or 504 plan, housing status, English learners, and free/reduced-price meal eligibility (FRM).

### School support plans/accommodations

CPS determines asthma status by 504 or IEP plan presence with asthma as an included diagnosis in one of these plans [46]. IEPs are for students whose asthma, other chronic conditions, and/or learning differences that impact educational performance such that a student requires specialized instruction. Often, other diagnoses prompt an IEP, and asthma accommodations are added to this plan [31].

## Results

### Descriptive statistics

#### School characteristics (Table 1)

**Table 1:** School and student characteristics based on pre-pilot enrollment years 2022–2023

		Phase 1	Phase 2	Total	*p* Value^†^
No. of schools*		306	211	517	
No. of students		159,714	106,349	266,063	
*School characteristics*					
Region					
	North	37 (12.1%)	20 (9.5%)	57 (11.0%)	0.41
	Northwest	47 (15.4%)	38 (18.0%)	85 (16.4%)	0.41
	West	66 (21.6%)	49 (23.2%)	115 (22.2%)	0.41
	Central	6 (2.0%)	10 (4.7%)	16 (3.1%)	0.41
	Southwest	52 (17.0%)	27 (12.8%)	79 (15.3%)	0.41
	Southeast	55 (18.0%)	36 (17.1%)	91 (17.6%)	0.41
	Far South	43 (14.1%)	31 (14.7)	74 (14.3%)	0.41
Grade level					
	Elementary School^‡^	253 (82.7%)	170 (80.6%)	423 (81.8%)	0.62
	High School	53 (17.3%)	41 (19.4%)	94 (18.2%)	0.62
School size					
	Median [q1, q3]	415 [266, 639]	395 [253, 616]	406 [263, 625]	0.44
Attendance rate					
	Median [q1, q3]	90% [87%, 92%]	89% [87%, 92%]	90% [87%, 92%]	0.30
*Student characteristics*					
Gender					
	Female	78,895 (49.4%)	52,279 (49.2%)	131,174 (49.3%)	0.23
	Male	80,718 (50.5%)	53,993 (50.8%)	134,711 (50.6%)	0.25
	Non-binary	99 (0.06%)	77 (0.07%)	176 (0.07%)	0.34
	Not reported	2	0	2	0.67
Race/ethnicity^¶^					
	Hispanic	74,468 (46.6%)	51,143 (48.1%)	125,611 (47.2%)	< 0.001
	Black, non-Hispanic	54,287 (34.0%)	31,148 (29.2%)	85,435 (32.1%)	< 0.001
	White, non-Hispanic	19,756 (12.4%)	14,750 (13.9%)	34,506 (13.0%)	< 0.001
	Asian or Pacific Islander	7,559 (4.7%)	6,657 (6.26%)	14,216 (5.3%)	< 0.001
	Two or more	2,675 (1.7%)	1,896 (1.8%)	4,571 (1.7%)	0.04
	Not specified	576 (0.4%)	445 (0.4%)	1,021 (0.4%)	0.02
	Native American	393 (0.2%)	310 (0.3%)	703 (0.3%)	0.03
Asthma**		8,561 (5.7%)	5,710 (5.37%)	14,271 (5.36%)	0.93
Food allergy		3,808 (2.4%)	2,819 (2.6%)	6,627 (2.5%)	0.08
Non-food allergy		263 (0.2%)	156 (0.1%)	419 (0.16%)	0.27
504		9,049 (5.7%)	6,200 (5.8%)	15,249 (5.7%)	0.08
IEP		24,158 (15.1%)	16,662 (15.7%)	40,820 (15.3%)	< 0.001
English learners^††^		35,415 (22.2%)	24,406 (23.0%)	59,821 (22.5%)	< 0.001
Free or reduced-price meals^‡‡^		112,181 (70.2%)	73,506 (69.1%)	185,687 (69.8%)	< 0.001
Unhoused^§§^		3,837 (2.4%)	2,436 (2.3%)	6,273 (2.4%)	0.06

*Neighborhood schools, excluding charter and private schools

^†^To identify differences between schools and students served between the two phases, the groups were compared by Chi-squared tests for categorical variables and Kruskal–Wallis tests for numerical variables

^‡^Elementary school includes Grades one through eight

^¶^Race/Ethnicity are reported together by CPS schools. Arranged in order of group size

**Asthma is counted by CPS as: number of students with current Asthma Action Plans on file with the school

^††^Students who indicate that they speak a language other than English at home are given the ACCESS English Language Proficiency exam. Students who do not pass this exam are defined by the Illinois State Board of Education as English Learners

^‡‡^Threshold for free or reduced-price meals: Income within 185% (reduced) or 130% (free) of the annual Federal Poverty Level

^§§^Unhoused includes students defined as homeless under the McKinney-Vento Act

Categorical variables (region and grade) were described as the number and percentage of schools in the 2022–2023 school year (SY) by phase. Numeric variables school size and attendance) were described by median value, first and third quartile.

#### Two-way associations with asthma prevalence

To explore the relationship between binary asthma diagnosis and student- and school-level characteristics, separate generalized estimating equations (GEE) logistic regression models were developed for each potential predictor. As a negligible number of records had missing gender or race and ethnicity values, each model used all available data from the 2021–2022 and 2022–2023 school years, treating school as a clustering variable. Estimated marginal mean asthma probability and odds ratios are reported with 95% confidence intervals and Sidak-adjusted *p*-values. Each odds ratio describes the relative odds of having an asthma diagnosis, with the largest subgroup treated as the reference level. For example, the FA odds ratio describes the ratio between the odds of asthma among those with a FA and the odds of asthma among those without a FA (Table 3).

#### Student characteristics

Student-level categorical variables (e.g., documented asthma diagnosis) were described as the number (percentage) of all students across all schools by phase and overall for the 2022–2023 SY.

To identify differences between schools and students served in the two phases, the groups were compared by Χ^2^ tests for categorical variables and Kruskal–Wallis tests for numeric variables. *p* < 0.05 suggests differences between groups.

#### Support plans/accommodations

Separate generalized estimating equation (GEE) logistic regression models were developed to estimate the probability of a student having two types of support plans—a 504 plan or an IEP—given their asthma status and other characteristics. Each model included multiple years of data and treated school as a clustering variable. Independent variables included student-level asthma status, race and ethnicity, gender, and grade level (a proxy for student age); school year; and school-level size, asthma rate, attendance rate, region, and IEP or 504 plan rate. To investigate differential effects of asthma status by race and ethnicity and gender, models also included two-way interactions between each of these variables and asthma. Estimated marginal probability of having each type of support plan by asthma status, race and ethnicity, and gender, averaged over levels of the other covariates, was calculated with 95% confidence intervals. Odds ratios with 95% confidence intervals and Sidak-adjusted *p*-values were calculated to describe the relative odds of each outcome by gender and then by race and ethnicity, stratified by asthma status.

### Sample (Table 1)

The stock inhaler program reached more than 266,063 students at the 517 district-run schools, consisting of 49.3% female and 50.6% male students that data was provided for.

### Asthma rates across school regions and grade levels (Table 2)

**Table 2:** Probability and odds of asthma by school and student characteristics

Variable	Level	Asthma Probability %	Odds ratio [95% CI]	*p* value
*School characteristics*				
Region				
	West	6.1		
	Central	4.8	0.79 [0.61, 1.02]	0.092
	Far South	4.8	0.78 [0.67, 0.90]	< 0.001
	North	4.8	0.78 [0.67, 0.91]	< 0.001
	Northwest	5.1	0.84 [0.73, 0.96]	0.005
	Southeast	5.3	0.86 [0.73, 1.02]	0.105
	Southwest	4.4	0.72 [0.62, 0.84]	< 0.001
Grade level				
	Elementary/ Middle School	4.6		
	High School	6.2	1.36 [1.26, 1.46]	< 0.001
School size				
	≤ 300	5.8		
	301–550	5.0	0.86 [0.78, 0.96]	0.003
	> 550	4.9	0.84 [0.76, 0.94]	< 0.001
School attendance				
	≤ 87	5.8		
	87–90	4.8	0.82 [0.75, 0.89]	< 0.001
	> 90	4.6	0.77 [0.71, 0.84]	< 0.001
*Student characteristics*				
Gender				
	Male	6.0		
	Female	4.1	0.66 [0.64, 0.69]	< 0.001
	Non-Binary	9.2	1.59 [0.89, 2.83]	0.138
Race/ethnicity				
	Hispanic^*^	5.1		
	American Indian	5.3	1.03 [0.67, 1.57]	1.000
	Asian, Hawaiian, or Pacific Islander	2.6	0.49 [0.39, 0.61]	< 0.001
	Black, Non-Hispanic	6.1	1.20 [1.11, 1.30]	< 0.001
	Two or more	5.1	1.00 [0.84, 1.18]	1.000
	White, Non-Hispanic	3.2	0.62 [0.55, 0.70]	< 0.001
Food allergy				
	No	4.3		
	Yes	38.0	13.70 [12.95, 14.50]	< 0.001
Non-food allergy				
	No	5.0		
	Yes	37.2	11.27 [9.40, 13.52]	< 0.001
504				
	No	3.2		
	Yes	37.7	18.40 [16.91, 20.02]	< 0.001
IEP				
	No	4.3		
	Yes	9.3	2.27 [2.17, 2.37]	< 0.001
English learners				
	No	5.4		
	Yes	3.8	0.70 [0.66, 0.74]	< 0.001
Unhoused				
	No	5.0		
	Yes	7.0	1.43 [1.30, 1.57]	< 0.001
Free or reduced-price meals				
	No	3.8		
	Yes	5.6	1.53 [1.45, 1.62]	< 0.001

*The reference for gender and race/ethnicity was based on the group with the highest total proportion of students. Male being the reference for gender and Hispanic being the reference for race/ethnicity. Further pairwise comparisons between groups were also conducted but not included in this table

Compared to students attending schools in the West region, students attending schools in the Far South (OR = 0.78 [0.67, 0.90], *p* < 0.001), North (OR = 0.78 [0.67, 0.91], *p* < 0.001), Northwest (OR: 0.84 [0.73, 0.96], *p* = 0.005), or Southwest regions (OR = 0.72 [0.62, 0.84], *p* < 0.001) were significantly less likely to have asthma.

As noted in Table 1, 81.8% of schools in the study sample were elementary schools (grades one through eight). High school students had significantly higher odds (OR:1.36 [1.26, 1.46], *p* < 0.001) of having asthma than elementary schoolers (Table 3).

**Table 3:** Probability and odds ratio of 504 and IEP by asthma status and other characteristics

			504 Probability	504 Odds Ratio [95% CI]	504 *p*-Value	IEP Probability	IEP Odds Ratio [95% CI]	IEP *p*-Value
Asthma								
	Gender							
		Male	36.6	1		32.1	1	
		Female	41.2	1.2 [1.1, 1.3]	< 0.001	21.2	0.6 [0.5, 0.6]	< 0.001
		Non-Binary	47.4	1.6 [0.5, 5.0]	0.631	13.4	0.3 [0.1, 1.6]	0.221
	Race/ Ethnicity							
		Hispanic	42.1	1		27.0	1	
		American Indian	31.9	0.6 [0.3, 1.4]	0.543	37.4	1.6 [0.7, 3.6]	0.478
		Asian, Hawaiian, or Pacific Islander	47.3	1.2 [0.9, 1.7]	0.403	11.5	0.4 [0.2, 0.5]	< 0.001
		Black, Non-Hispanic	32.7	0.7 [0.6, 0.7]	< 0.001	30.6	1.2 [1.1, 1.3]	< 0.001
		Two or more	43.9	1.1 [0.8, 1.5]	0.986	21.5	0.7 [0.5, 1.1]	0.193
		White, Non-Hispanic	41.6	1.0 [0.8, 1.2]	0.999	22.9	0.8 [0.7, 1.0]	0.009
No Asthma								
	Gender							
		Male	3.1	1		19.0	1	
		Female	2.6	0.8 [0.8, 0.9]	< 0.001	10.2	0.5 [0.5, 0.5]	< 0.001
		Non-Binary	12.1	4.3 [3.0, 6.1]	< 0.001	15.3	0.8 [0.5, 1.2]	0.312
	Race/ethnicity							
		Hispanic	2.7	1		15.2	1	
		American Indian	2.9	1.0 [0.7, 1.6]	0.999	16.2	1.1 [0.8, 1.4]	0.943
		Asian, Hawaiian, or Pacific Islander	2.4	0.9 [0.8, 1.0]	0.122	8.2	0.5 [0.4, 0.6]	< 0.001
		Black, Non-Hispanic	2.5	0.9 [0.8, 1.0]	0.017	14.6	1.0 [0.9, 1.0]	0.092
		Two or more	4.4	1.7 [1.4, 1.9]	< 0.001	11.2	0.7 [0.6, 0.8]	< 0.001
		White, Non-Hispanic	4.5	1.7 [1.5, 1.9]	< 0.001	12.2	0.8 [0.7, 0.8]	< 0.001

### Differences between pilot and ramp-up schools (Table 1)

There was no significant difference noted across phases of enrollment for region, school size, and attendance. There were statistically significant differences identified between phases for certain student characteristics. Statistically significant differences were found across phases for race and ethnicity (*p* < 0.001). The proportion of Hispanic students increased slightly from Phase 1 (46.6%) to Phase 2 (48.1%), and the proportion of Black, non-Hispanic students decreased from 34.0 to 29.2%. There was a significant increase in the proportion of students with an IEP between Phase 1 (15.1%) and Phase 2 (15.7%) (*p* < 0.001). The proportion of English Learners increased slightly from 22.2% in Phase 1 to 23.0% in Phase 2 (*p* < 0.001). A significant difference was observed in the percentage of students eligible for FRM, which decreased slightly from 70.2% in Phase 1 to 69.1% in Phase 2 (*p* < 0.001).

### Association between asthma rate with school size and attendance

Students attending schools with between 301 and 550 students enrolled had significantly lower odds (OR: 0.86 [0.78, 0.96], *p* = 0.003) of having asthma than students in schools with fewer than 300 students. Similarly, students attending schools with more than 551 students had significantly lower odds (OR: 0.84 [0.76, 0.94], *p* < 0.001) of having asthma than students attending schools with less than 300 students.

Compared to students attending schools with an average attendance lower than 87%, students attending schools with an average attendance between 87.1% and 90% had significantly lower odds of having asthma (OR: 0.82 [0.75, 0.89], *p* < 0.001), as did students attending schools with an average attendance over 90% (OR: 0.77 [0.71, 0.84], *p* < 0.001).

### Odds of asthma status by various student characteristics (Table 2).

Female students had significantly lower odds of having asthma compared to male students (OR: 0.66[0.64–0.69], *p* < 0.001). Hispanic students (reference group) had an asthma probability of 5.1%, similar to multi-racial students (OR: 1.00[0.84, 1.18], *p* = 1). Compared to Hispanic students, non-Hispanic Black students had the highest odds of asthma (OR:1.20[1.11, 1.30], *p* < 0.001), while non-Hispanic white students had lower odds (OR:0.62[0.55, 0.70], *p* < 0.001). Asian/Pacific Islander students had the lowest odds of asthma (OR:0.49[0.39, 0.61], *p* < 0.001).

Students with FA had higher odds of asthma (OR:13.7 [12.95, 14.50], *p* < 0.001) versus those without. Similarly, students with non-food allergies had higher odds of asthma (OR:11.27[9.40, 13.52], *p* < 0.001), than those without non-food allergies.

Students with any 504 plan had higher odds of asthma (OR:18.40[16.91, 20.02], *p* < 0.001) versus those without. This is likely due to the push for students with asthma to have a 504 plan, although not all students with asthma had an accommodation plan. Students with an IEP had higher odds of asthma (OR:2.27[2.17, 2.37], *p* < 0.001), than those without. English Learners had lower odds of asthma (OR: 0.70[0.66, 0.74], *p* < 0.001) compared to non-English Learners. Students receiving FRM had higher odds of asthma (OR:1.53[1.45, 1.57], *p* < 0.001) compared to those not receiving these meals. Unhoused students had higher odds of asthma (OR:1.43[1.30, 1.57], *p* < 0.001) relative to students with secure housing.

### Support plans by gender and race and ethnicity and asthma status (Table 3)

Female students with asthma had a 41.6% probability of having a 504 plan, compared to a 36.6% probability for male students with asthma. The odds of having a 504 plan were significantly higher for female students with asthma compared to male students with asthma (OR: 1.2 [1.1, 1.3], *p* < 0.001). Among female and male students without asthma, the probability of having a 504 plan was 2.6% and 3.1%, respectively, and the odds ratio was (OR: 0.8 [0.8, 0.9], *p* < 0.001).

When looking at IEP plans for students with asthma, males had a 32.1% probability of having an IEP plan, while female students had a 21.2% probability of having an IEP plan. The odds of having an IEP plan were significantly lower among females with asthma compared to males with asthma (OR: 0.6[0.5, 0.6], *p* < 0.001). The probability of having an IEP plan was 19% for male students without asthma and 10.2% for female students without asthma. Female students without asthma had half the odds of having an IEP plan (OR: 0.5 [0.5, 0.5], *p* < 0.001).

Black students without asthma had a 2.5% probability of having a 504 plan and a 14.6% probability of having a 504 plan. For Black students with asthma, the probability of having a 504 plan was 32.7% and the probability of having an IEP plan was 30.6%. When comparing race and ethnicity subgroups, Black students with asthma had lower odds of having a 504 plan (OR:0.7[0.6, 0.7], *p* < 0.001) but higher odds of having an IEP plan (OR: 1.2[1.1, 1.3], *p* < 0.001) compared to Hispanic students with asthma. White students with asthma had no significantly higher odds of having a 504 plan (OR: 1.0[0.8, 1.2, *p* > 0.999], but significantly lower odds of an IEP plan (OR: 0.8[0.7, 1.0], *p* = 0.009) compared to Hispanic students. A similar trend is noted for Asian students.

## Discussion

Our secondary data analysis of over 250,000 students reveals persistent asthma disparities across gender, race, school-level factors, and region within a large urban district. The current asthma rate in CPS differs from known neighborhood asthma prevalence and within the stock inhaler program intervention schools. Disparities also remain in asthma-related accommodations. These findings demonstrate the necessity for district-wide, multi-level interventions that improve access to comprehensive school-based asthma management [33]. Schools across the world, especially those with high asthma rates, limited access to medication among the student population, and longer emergency service response times may consider implementing asthma management practices like stock inhaler programming for emergency preparedness.

Asthma rate in CPS was lower than expected given the neighborhood asthma prevalence from the Chicago Health Atlas, but similar to other previous studies [13, 18]. The racial and sex disparities we discovered are similar to those observed on the national level [34]. Odds of asthma in CPS were highest among those with food allergies, a 504 and/or IEP, or FRM. Gupta’s 2008 study on childhood asthma prevalence in Chicago highlighted geographic variability in asthma rates. As Volerman [20] noted, it is still difficult to identify asthma cases within schools, leading to underdiagnosis and inadequate monitoring. By using these student-level variables that were associated with increased odds of asthma we could better pinpoint where to target resources. Resource allocation is needed to support the health of school-aged children so that they can remain in school and reach their potential. [7]. Better identification of cases and timely access to inhalers can improve overall quality of life for historically marginalized students with higher asthma prevalence. While universal access to inhalers is ideal, targeted interventions can ensure best resource allocation.

While both 504 and IEP plans are important for students with asthma, disparities and gaps in coverage remain. 504 or IEP plans are associated with better school performance and retention, but many either do not receive a plan or the plan does not accurately meet their needs [23, 35, 36]. This can be due to communication barriers, school nurses not being physically present in every school due to shortages, or limitations of caregiver knowledge and/or guidance about the process to design a plan. Studies suggest that support plans are often not initiated until school performance and/or attendance drops dramatically [37, 38].

Within CPS, females with asthma are more likely to have a 504 plan while males with asthma are more likely to have IEP plans. Students who are Asian/Pacific Islander, White, Multi-racial, and Hispanic are more likely to have 504 plans, while Black students are more likely to have IEP plans. Overall, the data shows that coverage with 504 and IEP plans remains low among students with asthma in CPS and may not serve as an accurate proxy for asthma rates. Without these support plans in place, students with asthma are at higher risk for poor school performance due to their health condition [35, 36]. Identification of subgroups who are less likely to receive support plans offers an intervention point for schools to ensure equitable access to education, which is listed under article 23 of the Convention on the Rights of the Child [39]. Nurses are critical advocates for students in the school setting to identify asthma cases and carry out management plans [37, 38, 40]. However, staffing, training, and access to medications are limiting factors [40, 41].

This data has several limitations, starting with underdiagnosis of asthma in the student population, therefore the estimated rate of asthma is likely underrepresenting the burden of asthma in CPS. In CPS, the number of 504 and IEP plans are used to assess asthma prevalence, and if not all students with asthma are covered by these plans, it remains difficult to capture asthma burden. Pilot schools were chosen based on high asthma rates, but this may not have been a selective upscale, given limited accuracy of asthma count. This data also does not include other conditions that may fall under 504 and IEP plans. Generalizability of these findings may be limited given the focus on CPS, but similar asthma-related issues are likely seen elsewhere [42, 43]. Finally, we were unable to perform multiple regression as the data was received in aggregate form, so we are unable to identify interactions between factors that influence asthma rates.

## Conclusion

This analysis of the pilot and upscale of the stock inhaler program in CPS reveals disparities in asthma prevalence and gaps in support plan coverage, which may be affecting the approach to stock inhaler distribution. Using school-based data to identify factors associated with asthma could allow for better case identification and serves as a target for interventions including stock inhaler distribution, staff and training needs, and support plan fulfillment. A randomized approach to upscale may serve as a better way to reach students who need inhalers and identify cases for further support plan implementation. Over time, better case identification and data-driven intervention has the potential to reduce the long-term impact of asthma on students and may serve as a model for other urban school systems.

## Data Availability

Data is available upon reasonable request from author Dr. Andrea A Pappalardo (email: apappa2@uic.edu, phone: 312-996-7119).
